# Embryos derived from donor or patient oocytes are not different for in vitro fertilization outcomes when PGT allows euploid embryo selection: a retrospective study

**DOI:** 10.1186/s40169-020-0266-1

**Published:** 2020-02-07

**Authors:** Elizabeth Schaeffer, Leonardo M. Porchia, Adina Neumann, Almena Luna, Tania Rojas, Esther López-Bayghen

**Affiliations:** 1 Laboratorio de Investigación y Diagnóstico Molecular, Instituto de Infertilidad y Genética, Ingenes, México City, Mexico; 2 Departamento de Toxicología, Centro de Investigación de Estudios Avanzados del Instituto Politécnico Nacional, Av. Instituto Politécnico Nacional 2508, Col. San Pedro Zacatenco, CP 07360 México City, Distrito Federal Mexico

**Keywords:** Aneuploidy, Preimplantation genetic diagnosis, Implantation

## Abstract

**Background:**

At our facilities, patients that received embryos using donor oocyte during in vitro fertilization (IVF), usually have had at least one failed attempt to produce at least one euploid embryo with their own oocytes; however, the current debate between using donor over patient oocytes remains inconclusive. We examined the aneuploidy rate and IVF clinical outcomes from embryos derived from either donor or patient oocytes.

**Methods:**

Retrospectively, 973 cycles were examined of patients who underwent a standard IVF protocol. Chromosomal content was determined using Pre-implantation Genetic Testing (PGT) by either microarray-comparative genomic hybridization or Next-generation sequencing from either Day 3 (blastocysts) or Day 5 (trophectoderm) embryo biopsies, respectively. Embryo implantation was confirmed by serum β-hCG (> 10 m IU/mL/Day 14), whereas clinical pregnancy by a fetal heartbeat (Week 6.5–8).

**Results:**

Embryos derived from donor oocytes presented with more monosomies than embryos derived from patient oocytes (41.2% vs. 25.4%, p < 0.05, respectively); however, only Trisomy 7 (0.4% vs. 2.3%, p < 0.05) and Trisomy in X (0.7% vs. 2.3%, p < 0.05) were significantly less present when compared to patient oocyte derived embryos. Interestingly, rates for embryo implantation (46.7% vs. 50.8%, p = 0.35), clinical pregnancy (38.5% vs. 43.1%, p = 0.30), and live birth (30.5% vs. 30.5%, p = 0.99) were similar for embryos derived from donor and patient oocytes. These results did not change when adjusted for the number of embryos implanted.

**Conclusion:**

Here, we show no significant differences in achieving pregnancy when using donor oocytes. Taking into consideration that aneuploidy rates are > 30% in embryos, independent of the oocyte origin, PGT should be recommended with donor oocytes as well.

## Background

Numerous factors affect the clinical outcomes of Assisted Reproduction, one of the most significant being aneuploid embryos [1]. An essential step in the in vitro fertilization (IVF) procedure is selecting high-quality embryos for uterine transfer, which is based on morphological assessment and cellular division rates [2, 3]. However, while these criteria are associated with improved IVF outcomes [2, 4], the overall IVF success rate remains low. Moreover, as the patient’s age increases, the success of IVF outcomes, such as implantation, clinical pregnancy, and live birth, diminish [5]. Therefore, in many countries, current guidelines suggest for women of advanced age to complement IVF with Pre-implantation Genetic Diagnosis Testing (PGT) to improve the selection of genomically euploid, high-quality embryos [6] or possibly using donor oocytes. Patients receiving donor oocytes in IVF usually have had at least one or more failed attempts to conceive with their own oocytes.

Hormones used during ovarian-stimulation can increase the aneuploidy rates in donors [7]. These hormones are shown to affect protein synthesis, cellular division, and membrane fluidity and structure—key factors associated with embryo implantation [8]. Moreover, the aneuploidy rate of donor oocyte derived embryos, which ranges between 20 and 60% [9], are similar to patient oocyte derived embryos, when the patient’s age is above 27 years [10]. Interestingly, Munne and colleagues demonstrated that the euploidy rate is associated with IVF clinics, thus indicating the procedural, material, and professional experience and preferences lead to a clinic effect [9].

Most women of advanced age experience a decrease in their ovarian reserve with changes in hormonal dysfunction due to natural biological aging mechanisms [11]. The combined effect of increased aneuploidy rates and diminished ovarian response manifests as an increased proportion of IVF cycles where no euploid embryos are detected [12, 13]. Therefore, it has been posited that oocytes collected from donors compared to the patient does significantly improve IVF outcomes [14], with the main reason for using donor oocytes is the failure of producing sufficient oocytes from the patient.

Here, we assess the quality of embryos produced from donor oocytes compared with patient oocytes, with respect to the aneuploidy rate, type of aneuploidies, the chromosomes which are more affected by monosomy or trisomy, and we determined if embryos produced using donor oocytes improved IVF clinical outcomes in women from Mexico City.

## Materials and methods

### Study participants

**Fig. 1:**
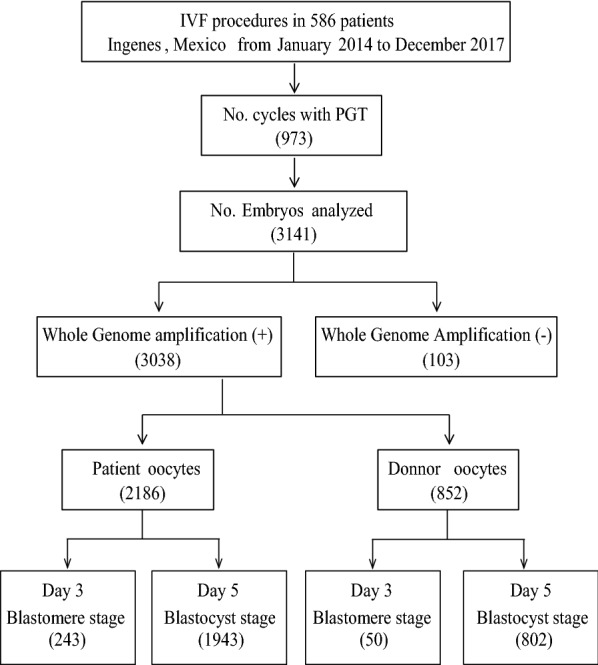
Flow chart of embryo selection

From January 2014 to December 2017, clinical data from women undergoing IVF cycles were examined for this retrospective study. To be included in this study, either Day 3 or Day 5 embryo biopsies had to be analyzed by either microarray-comparative genomic hybridization (aCGH) or Next-generation sequencing (NGS) for all 23 chromosome pairs. Furthermore, all oocytes were fresh and fertilized using intracytoplasmic sperm injection (ICSI). A participant was removed if any data, with regards to PGT and IVF outcomes, were missing. Figure 1 shows a graphic and comprehensive explanation of the study organization for patient sampling.

### IVF

For patient oocytes, all patients were subjected to a controlled ovarian stimulation for 10 to 20 days with gonadotrophin-releasing hormone (GnRH) agonists and antagonists. Briefly, the controlled ovarian stimulation protocol consisted of administering a daily dose of a GnRH antagonist (0.25 mg/day Cetrorelix, Cetrotide, Merck México, or 0.25 mg/day Ganirelix acetate, Orgalutran Laboratorio Msd) in the luteal phase after menses. Gonadotropins were administered in variable doses (with a minimal daily dose of 300 IU), dependent on the patient’s age and ovarian responsiveness, with further adjusting according to serum estradiol (E_2_) levels and vaginal ultrasound measurements of the follicular diameter obtained every 2 or 3 days. Stimulation was prolonged until the mean diameter of the leading follicles was > 18 mm. Recombinant human Chorionic Gonadotropin (hCG) (Choragon 1000 IU, Ferring) was administered, and oocyte retrieval was conducted 36 h after the administration of hCG with ultrasound guidance.

For donor selection, a detailed clinical file with gynecological data describing regular cycles and proven fertility was developed. The donors had to meet the following criteria: age range between 18 and 32 years; BMI between 19 and 29 kg/m^2^; non-smoker and non-drug user; no tattoos or piercings in the last 6 months; no reproductive disorders/abnormities; no family history of inheritable genetic disorders proven by a karyotype analysis; no diagnosis of cancer; without infection (HIV, Hepatitis B and C, VDRL, rubella, and Toxoplasma); and the donor’s personality and psychopathology was assessed by the Minnesota Multiphasic Personality Inventory. Fertility was proven by a previous pregnancy with a healthy baby delivered and sufficient oocyte count number. Most donors had previously donated oocytes that resulted in successful pregnancies. For oocyte donors, ovarian stimulation started on the second or third day of the menstrual period with 225–300 units of Merional (CORNE) for 10–12 days. Once serum E_2_ levels indicated the stimulation had been completed, ovulation was induced with Gonapeptyl (0.10 mg/1 mL Ferring) or Ovidrel (6.500 UI Merck).

In all cases, ovarian response was assessed by measuring serum E_2_ levels every other day, and follicular development was evaluated by ultrasound examination. Oocyte retrieval was conducted after hCG, Gonapeptyl, or Ovidrel administration with ultrasound guidance. All 14–18 mm follicles were aspirated, and the oocytes were retrieved: patient using their own oocytes, between 6 and 14 (average = 10.5 ± 2.5), and donors, between 7 and 42 (average 22.5 ± 8.1). The number and quality of retrieved oocytes were assessed using morphological parameters [granulosa expansion, oocyte maturity (MI, MII, and VG), quality of the cytoplasm, zona pellucida, and polar body].

The partner’s sperm were prepared by density gradient centrifugation. The fresh oocytes (patient or donor) were all inseminated by ICSI, and fertilization was judged by the formation of two pronuclei 19 h after insemination. Embryos were cultured in Global Total for Fertilization media (Cat # LGGT-30, LifeGlobal) and incubated at 37 °C in 8% CO_2_, 5% O_2_, and 87% N_2_. Embryo morphological parameters evaluated were weighed into a matrix to rate each embryo, with the sum of values obtained on a scale of 0 (low quality) to 12 (high quality). Embryo selection was done according to the embryo morphological assessment, using the criteria established by the Istanbul Consensus Workshop on Embryo Assessment [11]. For Day 3 embryos biopsies (blastocysts), embryo transfers were done on Day 5 (fresh cycle); however, for Day 5 embryos biopsies (trophectoderm), the resulting blastocysts were cryopreserved by vitrification and thawed for transfer in a new natural cycle with endometrial preparation. An embryologist monitored and recorded all information about fertilization rates, embryo development, and embryo morphology for each oocyte.

### Day 3 and Day 5 embryo biopsy

For Day 3 embryos, we utilized the S-biopsy method to isolate a blastomere [15]. Briefly, a Hamilton Thorne ZILOS-tk laser (1460 nm, 300 mW) was used to create a thin funnel in the zona pellucida adjacent to the desired blastomere. Next, the blastomere was extracted by aspirating the whole embryo into a 140-µm stripper capillary micropipette, leading to the ejection of the blastomere. The blastomere was then placed into a 0.2-µL PCR tube.

For Day 5 embryos (expanded blastocyst stage containing 50 to 150 cells), a laser was used to create a thin funnel in the zona pellucida on the opposite side to the inner cell mass. Blastocysts were incubated for a further 2–3 h to allow blastocoele expansion and herniation of the trophectoderm cells from the zona. Afterward, the embryo was placed into 20-µL of Ca^2+^/Mg^2+^-free bicarbonate buffered G-PGD medium (cat #10074, Vitrolife). Applying gentle suction with the biopsy pipette (MBB-FP-SM-35, Origio, Malov, Denmark), the trophectoderm cells were encouraged to herniate from the zona. Four to five trophectoderm cells were dissected from each of the blastocysts using four laser pulses of 3-min duration, and a total of 10–15 cells were retrieved, washed, and placed into a 0.2-µL PCR tube.

### Whole genome amplification (WGA), aCGH, and NGS

All of Day 3 embryos were analyzed with aCGH. For aCGH, each biopsy was amplified using the SurePlex amplification system (BlueGnome, San Diego, CA, USA) according to the manufacturer’s instructions. aCGH was carried out using the 24 Sure V3 microarrays (Illumina, San Diego, CA, USA) and the protocol described by Fragouli [16, 17]. The amplified DNA was fluorescently labeled (Fluorescence Labelling System, BlueGnome). The samples were co-precipitated, denatured, and analyzed by array hybridization. The hybridization time was 16 h. A laser scanner (InnoScan 710, Innopsys, Carbonne, France) was used to excite the fluorophores and read the hybridization images. The hybridization images were stored in TIFF format and analyzed by the BlueFuse Multi-Analysis software (BlueGnome), using the criteria and algorithms recommended by the manufacturer. With this approach, it was possible to determine the chromosome constitution of each embryo.

All of Day 5 embryos were analyzed with NGS. For NGS, each biopsy sample was amplified using the SurePlex DNA amplification system (Illumina Inc.) according to the manufacturer’s instructions. WGA products were quantified using Qubit 3.0 Fluorometer (Life Technologies). The library preparation was carried out with the VeriSeq PGS Library Prep Kit (Illumina Inc.). DNA ‘indexing’ was performed to simultaneously analyze samples from different embryos, using the Nextera XT 96-Index Kit (Illumina, Inc.). For library preparation, 5 µL (0.2 ng/µL) of each WGA product from each sample was tagmented (tagged and fragmented) by the VeriSeq PGS transposome using the manufacturer’s protocol and neutralized by adding 5 µL of neutralization buffer. The tagmented DNA was amplified with index 1 primers (N701to N712) and index 2 primers (S503 and S504) to become the NGS library via a limited cycle PCR program. Each sample’s NGS library was purified to remove short fragments and primers. Finally, NGS libraries were pooled, denatured with HT1, and loaded to the VeriSeq PGS (Illumina Inc.) sequencing cartridge following the manufacturer’s protocol. NGS library was sequenced with a MiSeq apparatus. Sequencing data were generated by MiSeq Reporter Software. Chromosome content was determined, as indicated above.

### Endometrial preparation and embryo transfer

Transfer of cleavage-stage biopsied embryos (Day 3) took place on Day 5 (fresh transfer, same cycle). Transfer of trophectoderm biopsied embryos (Day 5) were thawed and transferred in a controlled endometrial development cycle into the uterus, free of gonadotropin stimulation. Clinical decisions about which and how many embryos to transfer were determined by the Physician and Specialist in Reproductive Medicine with the patient’s approval. Embryo implantation was confirmed on Day 14 by β-hCG serum levels > 10 mIU/mL or the presence of a fetal heartbeat by ultrasound at 6.5 to 8 weeks. All the patient’s demographics, IVF cycle, PGT results, implantation rate, and IVF outcomes (pregnancies and miscarriages) were recorded by the Specialist.

For endometrial preparation, patients were taking Microgynon (Levonorgestrel 0.15 mg and Estradiol 0.03 mg, Bayer) starting on the second day of their previous cycle for a period of 21 days. For 15 days, patients are examined to determine the absence of residual follicles. Triptorelin Acetate (3.75 mg, Gonapeptyl Depot Ferrin, or Pamorelin Actuamed) was then applied intramuscularly on Day 17 of their cycle. After 3 to 5 days, the patient stopped taking the contraceptive, and the menstrual cycle started. If the endometrium showed to be smaller than 4 mm and no residual follicles were present, endometrial preparation began during Day 3. Endometrial preparation was carried out with the application of Evorel 50 (150 micrograms/subcutaneous/every 48 h), and the luteal phase support was carried with Utrogestan (300 mg/day) until the serum E2 concentration remained constant. Afterward, Gonapeptyl Depot was applied (2 mg every 12 h for 4 days increasing the dosage to 2 mg every 8 h). During Day 10 of endometrial preparation, endovaginally ultrasound was performed to assess the characteristics and thickness of the endometrium. Endometrium must be trilaminar and between 8 and 12 mm thick in order to schedule embryo transfer. Lastly, progesterone was applied endovaginally (3 doses, 300 mg each).

### Statistical analysis

All analyses were carried out with the Statistical Package for the Social Sciences software (SPSS v22.0, Chicago, IL USA). The normality of the data was assessed by the Shapiro–Wilk test. Differences between categorical data were assessed with the Chi Square test. Homogeneity of the variances in parametric data was determined with Levene’s test. Differences between groups were determined with ANOVA with a post hoc Dunnett T3 or Bonferroni test, according to their homogeneity. For non-parametric data, differences between groups were determined with the Kruskal–Wallis test with a post hoc Dunn’s test. The Pearson correlation coefficient (r) was used to determine the association between variables. Multinomial logistic regression was used to determine the odds ratio (OR) and 95% confidence interval (95%CI), evaluating the level of association. p-values < 0.05 (two-tailed) were considered statistically significant.

## Results

### Characteristics of the cohort

Five hundred eighty-six patients agreed to participate in this study, totaling 973 cycles. 3,141 embryos were collected for analysis, biopsy, and WGA. 103 embryos (3.28%) failed to amplify. Of the donor and patient embryos, 5.87% and 11.12% were blastomere biopsies (Day 3), respectively. When the data were categorized by biopsy day, and thus by the method of detection (aCGH or NGS), there was no difference in the results for any endpoint assessed (data not shown). The selection of the participants is shown in Fig. 1. The most common reason to undergo PGT was advanced age (38.62%), followed by a low response to ovarian stimulation (15.02%), sex selection for medical reasons (8.57%), and repeated failed implantation (8.57%). There was a significant decrease in the number of oocytes collected from women as their aged increased (r = − 0.321, p < 0.001, Table 1). A similar result was observed for the number of fertilized eggs (r = − 0.305, p < 0.001) and the number of embryos on Day 3 (r = − 0.235, p < 0.001). However, increases in age were not associated with changes in fertilization rates. Cohort characteristics are summarized in Table 1.

**Table 1:** Characteristic of the participants

	Age groups, own oocytes						Donor oocytes
Age category	≤ 29^a^	30–34^b^	35–37^c^	38–40^d^	41–43^e^	≥ 44^f^	Donor age: 18–32^g^ BMI: 23.6 ±
Size (n)	45	112	144	206	167	60	236
Recipient age (years)^h^	26.4 ± 2.7^b,c,d,e,f,g^	32.4 ± 1.3^a,c,d,e,f,g^	36.1 ± 0.7^a,b,d,e,f,g^	39.0 ± 0.8^a,b,c,e,f,g^	41.8 ± 0.8^a,b,c,d,f,g^	45.1 ± 1.6^a,b,c,d,e,g^	42.0 ± 4.6^a,b,c,d,f^
Recipient BMI (kg/m^2^)	23.6 ± 3.5	25.0 ± 4.1	24.7 ± 4.0	24.4 ± 3.6	24.8 ± 4.0	25.2 ± 3.5	25.3 ± 3.9
Oocyte collected (n)	18.1 ± 9.1^d,e,f^	16.7 ± 9.3^d,e,f^	15.6 ± 8.3^d,e,f^	11.8 ± 6.4^a,b,c,g^	11.3 ± 6.9^a,b,c,g^	8.8 ± 6.6^a,b,c,g^	15.7 ± 6.4^d,e,f^
Oocyte fertilized (n)	13.4 ± 6.4^d,e,f^	12.6 ± 7.9^d,e,f^	11.5 ± 6.9^d,e,f^	8.7 ± 5.0^a,b,c,g^	8.3 ± 5.6^a,b,c,g^	6.5 ± 4.8^a,b,c,g^	11.2 ± 5.0^d,e,f^
Embryos (n)	8.3 ± 6.1^d,e,f^	8.6 ± 7.3^d,e,f,g^	7.2 ± 5.7^d,e,f^	5.4 ± 4.0^a,b,c^	5.3 ± 4.3^a,b,c^	4.2 ± 3.6^a,b,c,g^	6.5 ± 4.3^b,f^
Fertilization rate (%)	73.2 ± 17.3	69.8 ± 18.3	71.4 ± 19.2	69.5 ± 18.4	68.5 ± 21.9	75.2 ± 22.2	69.0 ± 17.4
Aneuploidy rate (%)	27.5 ± 30.9^d,e,f^	37.5 ± 30.7^d,e,f^	37.6 ± 32.5^d,e,f^	56.9 ± 35.4^a,b,c,e,f,g^	67.9 ± 36.5^a,b,c,d,f,g^	87.5 ± 24.9^a,b,c,d,e,g^	27.5 ± 26.9^d,e,f^
Cycles with no euploid embryos (%)	1.8^e,f^	4.6^e,f^	7.3^d,e,f^	26.9^b,c,e,f,g^	34.7^a,b,c,d,f,g^	20.1^a,b,c,d,e,g^	4.6^d,e,f^

Values are mean ± standard error. Significance was determined by one-way analysis of variance (ANOVA) followed by a Bonferroni or Dunnett’s T3 post hoc test

*BMI* Body-mass index

^a^Significant difference vs. ≤ 29 group, p < 0.05 (two-tailed)

^b^Significant difference vs. 30–34 group, p < 0.05 (two-tailed)

^c^Significant difference vs. 35–37 group, p < 0.05 (two-tailed)

^d^Significant difference vs. 38–40 group, p < 0.05 (two-tailed)

^e^Significant difference vs. 41–43 group, p<0.05 (two-tailed)

^f^Significant difference vs. ≥44 group, p<0.05 (two-tailed)

^g^Significant difference vs. donor group, p<0.05 (two-tailed)

^h^Three patients did not indicate their age

### Age-associated aneuploidies rates

High-quality embryos were assessed for aneuploidies using aCGH or NGS. As expected, the aneuploidy rate was highly correlated with maternal age (r = 0.405, p < 0.001, Table 1). There was a 2.19-fold increase in the aneuploidy rate between the youngest age group (≤ 29 years old) and the oldest age group (≥ 44 years old). There was no difference in respect to the aneuploidy rate between the donors and the comparable age group (patients ≤ 29 years old); however, there was a significantly higher overall aneuploidy rate for embryos from patient oocytes compared to donor oocytes (50.9% vs. 27.9%, respectively, p < 0.001, Table 2). The most common abnormality was a gain or a loss of one chromosome (53.8%, Table 2); this was independent of the source of the oocyte, patients or donors (50.7% vs. 68.1%, respectively). The most frequent monosomy was the loss of Chromosome 22, followed by Chromosome Y and Chromosome 16 (Additional file 1). The most frequent trisomy was Trisomy 16, followed by Trisomy 20, 21, and 22. Of the 302 dual abnormalities, 113 were a gain of two chromosomes, 60 were a loss of two chromosomes, and 129 were a gain and loss of one chromosome each. 322 embryos were determined to have multiple abnormalities: 3 (40.99%), 4 (22.98%), 5 (9.63%) and ≥ 6 (26.4%). Using multinomial logistic regression, we determined that age was shown to correlate with increased prevalence of monosomy 9, 11, 12, 13, 15, 16, 17, 18, 19, 20, 21 and 22, as well as trisomy 1, 3, 9, 11, 12, 13, 14, 15, 16, 17, 18, 20, 21 and 22 (Table 3, p < 0.05). There was no difference in the frequencies between the donors and patients ≤ 29 years old for a loss of a chromosome; however, only for Trisomy 7 and Trisomy X were patients ≤ 29 years old associated with an increased frequency (Additional file 1, p < 0.05).

**Table 2:** Distribution of types of aneuploidies

	Patient/donor oocytes	Patient oocytes		Donor oocytes
Age range	≥ 18	≥ 18	≤ 29	18–32
Total embryos	3038	2186	172	852
Abnormal embryos	1351 (44.5%)	1113 (50.9%)	46 (26.7%)	238 (27.9%)
Monosomy	381 (28.2%)	283 (25.4%)	15 (32.6%)	98 (41.2%)*
Trisomy	346 (25.6%)	282 (25.3%)	13 (28.3%)	64 (26.9%)
Dual	302 (22.4%)	266 (23.9%)	11 (23.9%)	36 (15.1%)*
Multiple	322 (23.8%)^a^	282 (25.3%)^b^	7 (15.2%)^c^	40 (16.8%)^d,^*

*Indicates a significant difference (p < 0.05, chi-2 test) in the rate of chromosome abnormalities between embryos from patient oocytes and donor oocytes. ** indicates a significant difference (p < 0.05, chi-2 test) in the rate of chromosome abnormalities between embryos from patient oocytes and donor oocytes

^a^Twenty-six embryos had completely abnormal chromosomal profiles

^b^Twenty-two embryos had completely abnormal chromosomal profiles

^c^Zero embryos had completely abnormal chromosomal profiles

^d^Four embryos had completely abnormal chromosomal profiles

**Table 3:** Risk associated with gain or loss of a chromosome

Chromosome	Gain^a^	Loss^a^
1	1.13 (1.04–1.23)*	0.96 (0.90–1.02)
2	1.08 (1.00–1.16)	1.01 (0.94–1.09)
3	1.15 (1.04–1.27)*	1.13 (1.00–1.29)
4	1.04 (0.97–1.12)	1.00 (0.92–1.09)
5	1.04 (0.96–1.13)	1.00 (0.93–1.08)
6	0.99 (0.94–1.05)	1.00 (0.91–1.11)
7	1.03 (0.97–1.10)	1.13 (0.99–1.28)
8	1.07 (0.98–1.17)	1.06 (0.97–1.15)
9	1.11 (1.04–1.18)*	1.12 (1.04–1.21)*
10	1.08 (0.99–1.13)	1.08 (0.98–1.18)
11	1.14 (1.06–1.24)*	1.24 (1.11–1.39)*
12	1.16 (1.07–1.25)*	1.21 (1.08–1.35)*
13	1.06 (1.00–1.13)*	1.10 (1.01–1.18)*
14	1.14 (1.06–1.23)*	1.08 (1.00–1.17)
15	1.17 (1.10–1.24)*	1.15 (1.09–1.23)*
16	1.08 (1.04–1.13)*	1.08 (1.02–1.14)*
17	1.19 (1.10–1.30)*	1.18 (1.04–1.34)*
18	1.07 (1.00–1.13)*	1.13 (1.05–1.22)*
19	1.04 (0.99–1.09)	1.18 (1.09–1.27)*
20	1.19 (1.11–1.26)*	1.13 (1.05–1.22)*
21	1.07 (1.03–1.12)*	1.14 (1.07–1.22)*
22	1.10 (1.05–1.15)*	1.20 (1.14–1.28)*
X	1.04 (0.99–1.10)	1.02 (0.96–1.10)
Y	0.91 (0.82–1.01)	1.05 (0.99–1.11)

*Indicates a significant result (p < 0.05, two-tailed)

^a^Values are odds ratios (OR) and 95% confidence intervals, which were calculated using multinominal logistic regression

### No difference in IVF clinical outcomes between embryos derived using either patient or donor oocytes

Embryos were implanted in only 592 cycles; the remainder had no viable euploid embryos, or their embryos were frozen and never implanted. Of the 592 cycles, 190 used embryos that were generated from donor oocytes. When analyzed as a cohort or by the number of embryos transferred, there was no statistical difference in the implantation rate, clinical pregnancy, or the live birth rate (Table 4). As indicated in Table 1, patients age ≤ 37 years old and ≥ 38 years old were similar in respect to the number of oocytes collected, the number of oocytes fertilized, the number of embryos collected, the fertilization rate, and the aneuploidy rate; therefore, all these patients were categorized by age into 2 groups. For recipients ≤ 37 years old (n = 246), only 19 cycles (< 10%) were from donor oocytes, demonstrating the preference for donor oocytes to be used with older women. Nonetheless, there was no difference in the implantation rate, clinical pregnancy, or the live birth rate as a whole or stratified by the number of implanted embryos. For recipients ≥ 38 years old (n = 346), in which 171 received embryos using donor oocytes, again, there was no difference for clinical outcomes. As expected, higher success rates for clinical outcomes were achieved with the transfer of more than one embryo.

**Table 4:** Comparison of clinical outcomes in recipients of embryos derived by donor or patient oocytes

Clinical outcomes	N^a^	Total		p^b^	Age ≤ 37		p^c^	Age ≥ 38		p^d^
		Patient oocytes (%)	Donor oocytes (%)		Patient oocytes (%)	Donor oocytes (%)		Patient oocytes (%)	Donor oocytes (%)	
Implantation^e^	1–3	188/402 (46.7)	97/190 (50.8)	0.35	117/227 (51.5)	10/19 (52.6)	0.93	71/175 (40.6)	87/171 (50.9)	0.06
	1	46/163 (28.2)	9/40 (22.5)	0.47	13/52 (25.0)	1/5 (20.0)	0.80	33/111 (29.7)	8/35 (22.9)	0.43
	2	111/192 (57.8)	65/109 (59.6)	0.76	80/143 (55.9)	6/10 (60.0)	0.80	31/49 (63.3)	59/99 (59.6)	0.67
	3	31/47 (66.0)	23/41 (56.1)	0.34	24/32 (74.0)	3/4 (75.0)	0.99	7/15 (46.7)	20/37 (54.1)	0.63
Clinical pregnancy^f^	1–3	155/402 (38.5)	82/190 (43.1)	0.30	96/227 (42.3)	9/19 (47.4)	0.67	59/175 (33.7)	73/171 (42.6)	0.09
	1	37/163 (22.7)	9/40 (22.5)	0.98	10/52 (19.2)	1/5 (20.0)	0.97	27/111 (24.3)	8/35 (22.9)	0.86
	2	93/192 (48.4)	53/109 (48.6)	0.98	68/143 (47.6)	5/10 (50.0)	0.88	25/49 (51.0)	48/99 (48.5)	0.77
	3	25/47 (53.2)	20/41 (48.8)	0.68	18/32 (56.3)	3/4 (75.0)	0.47	7/15 (46.7)	17/37 (45.9)	0.96
Live birth^g^	1–3	113/371 (30.5)	50/164 (30.5)	0.99	68/208 (32.7)	7/17 (41.2)	0.48	45/163 (27.6)	43/147 (29.3)	0.75
	1	27/153 (17.6)	5/38 (13.2)	0.51	7/49 (14.3)	1/5 (20.0)	0.73	20/104 (19.2)	4/33 (12.1)	0.35
	2	69/172 (40.1)	33/93 (35.5)	0.46	48/127 (37.8)	3/8 (37.5)	0.98	21/45 (46.7)	30/85 (35.3)	0.21
	3	17/46 (37.0)	12/33 (36.4)	0.96	13/32 (40.6)	3/4 (75.0)	0.19	4/14 (28.6)	9/29 (31.0)	0.87

Values are number positive/total number (percentage)

^a^Number of embryos transferred

^b^Difference between patient oocytes and donor oocytes were determined by Chi-2 test for the complete cohort

^c^Difference between patient oocytes and donor oocytes were determined by Chi-2 test for subjects ≤ 37 years

^d^Difference between patient oocytes and donor oocytes were determined by Chi-2 test for subjects ≥ 38 years

^e^Positive implantation was determined by β-hCG serum levels > 10 mg/dL at Day 14 after transfer

^f^Positive clinical pregnancy was confirmed by the presence of a fetal heartbeat at week 6–8 (ultrasound)

^g^Positive pregnancy was defined as a live birth

## Discussion

For women of advanced age or younger women that suffer from failed IVF treatments, it is suggested that donor oocytes may present with benefits over the use of their own oocytes. However, the data supporting this claim is conflicting [18]. Here, we show that, with respect to IVF clinical outcomes, embryos from donor oocytes were not superior to embryos developed using patient oocytes. This does posit that for many patients that suffer failed IVF outcomes, once a euploid embryo is selected, alternative factors, such as endometrium quality, female lifestyle, and others, are more likely to affect the IVF outcomes [19].

One of the most prevalent reasons for unsuccessful IVF procedures is implantation failure [20]. The reasons for failed implantations range from an unreceptive endometrium, uterine pathologies, to severely aneuploid embryos. Mainly, embryos are selected based on morphological assessment, which can be accompanied by PGT to detect aneuploidy status. However, the detection of aneuploid embryos and discarding them remains controversial (for review see [21]). Depending on the type of aneuploidy and its severity, the *Preimplantation Genetic Diagnosis International Society* has provided guidelines for selecting embryos with good potential for implantation [22, 23]. Here, we determined that 44.5% of the high-quality embryos examined were aneuploid, and 20.5% presented multiple chromosome abnormalities and would be discarded. The typical donor is fertile, young women, who are more likely to have low chromosome abnormalities [24, 25]. Interestingly, using donor oocytes did result in fewer aneuploidy embryos, with most abnormalities being monosomies. This does suggest that the use of donor oocytes could be beneficial. Thus, women with a low response or few euploid embryos may consider using donor oocytes.

Aneuploidy rates have been associated with increasing maternal age [26]. Here, we determined that, for Mexicans, certain chromosomes are significantly associated with a loss of one chromosome (9, 11, 12, 13, 15, 16, 17, 18, 19, 20, 21 and 22) or a gain of one chromosome (1, 3, 9, 11, 12, 13, 14, 15, 16, 17, 18, 20, 21 and 22). Therefore, older women may elect to use a donor’s oocytes to produce a viable embryo. However, a question that remains is there a difference between donor aneuploidy distribution and patient aneuploidy distribution. Here, we determined that, except for chromosomes 7 and X in which the donors had a lower frequency, there was no difference between the donor population and the comparable age group (patients ≤ 29 years old). This does support the use of donor oocytes for IVF procedures for women of advanced age, at least > 37 years, according to this study.

It is worthy to note that the embryo cohorts in this study were considered high-quality embryos by morphological assessment. However, hormone treatment, maternal age, and IVF procedural techniques can affect aneuploidy rates and embryo quality [3]. Therefore, we examined if the source of the oocyte affected the clinical outcomes. Interestingly, once euploid embryos were selected for transfer, there was no difference in using donor oocytes over patient oocytes concerning embryo implantation, embryo development to clinical pregnancy, and live birth. Moreover, these results were independent of maternal age.

Single embryo transfers are discouraged under certain circumstances: previous failed IVF procedures, recurrent miscarriages, or a history of genetic abnormalities. To increase the probability of implantation and live births, clinicians usually transfer two or three embryos; however, this does increase the potential of multiple births, which could be an unwanted result. There was a marked improvement in clinical outcomes using two or three implanted embryos over one embryo for women ≥ 38 years old, independent of the origin of the oocyte. For women ≤ 37 years, using two or three implanted embryos did improve clinical outcomes, but this could not be confirmed for the patients receiving embryos using donor oocytes due to few samples. This does speculate that the belief of using donor oocytes, with respect to age, improves IVF outcomes is flawed.

Two concerns with the cohort are that for females receiving oocytes from donors, the pregnancy rate was around 50%, and the aneuploidy rate was 25%. This could be due to unidentified confounding variables, such as endometrial receptivity, quality of sperm, the severity of inflammation due to the allogeneic embryo, level of insulin resistance and/or serum adipokines, miscarriage history of the embryo recipient, or other idiopathic reasons [19, 27]. Some of this data was collect for most of the patients; however, these external factors were not the focus of the manuscript and should be analyzed in future studies with a specific focus on these factors. Moreover, other studies and reports, in which the patient cohort was made up of only oocyte donors, the pregnancy rate was around 50% [28, 29]. Also, the aneuploidy rate could be around 25%, and, depending on the age of the sperm donor, exceed 60% [9, 10, 30]. Therefore, we believe that the oocyte donor group does represent the typical donor make-up of Central Mexicans.

Here, we demonstrated that certain chromosomes had a higher propensity to develop into monosomies and trisomies. A question that is posited is what are the factors that are associated with these abnormalities, such as chromosome length or gene count; moreover, does maternal age augment this association? Indeed, we found that smaller chromosomes were associated with an increased risk of developing monosomies or trisomies. Franasiak and colleagues did demonstrate that differences in aneuploidy rates are found when chromosomes are grouped by structural cytogenetic classification systems [31]. It was seen with both the classic karyotype groupings as well as the metacentric, submetacentric, and acrocentric designations. While these groupings are morphologic in nature and do not necessarily indicate specific function, it is of considerable biological interest that differences exist between these groups. According to Franasiak and colleagues, metacentric was 1.6 times and acrocentric 2.3 times more likely to have errors [31]. Here, the most common chromosomes which exhibited aneuploidies were comprised of both the short and medium acrocentric chromosomes (chromosome 13, 14, 15, 21, 22), which would result in failed IVF cycles, either at the embryo implantation stage or during fetal development. There was also an age-related increase in the proportion of errors that occur with acrocentric chromosomes relative to other types of chromosomes. Additionally, these particular chromosomes exhibited disproportionately higher aneuploidy rates with advancing age. As suggested by Hassold and colleagues, perhaps the process of crossing over during pachytene and separation during diplotene puts these particular chromosomes at risk [32]. Moreover, the biologic machinery governing this process appears to be susceptible to the aging process, associated with less fidelity over time.

Mosaicisms also present another concern affecting IVF outcomes [33]. With recent developments, PGT detection of embryonic mosaicisms has become possible [34], and many platforms can now detect embryonic mosaicisms. Mosaicisms result in different genotypes as a function of mitotic error, such as anaphase lag or chromosome non-disjunction [35]. The presence of unidentified mosaicisms may be the reason why certain euploid embryo transfers resulted in implantation failure or spontaneous miscarriages [36]. Although mosaicisms are now becoming a well-accepted phenomenon in preimplantation diagnosed embryos, its implications for PGT require more attention. Critical sources of variation in mosaicism detection may be a result of different platforms as diagnostic tools that may cause different levels of artifacts, the various genomic amplification systems employed, or the fact that embryos are being analyzed at different stages of development [37]. Nevertheless, the origins, severity, and consequences of mosaicisms and the effect on embryo implantation rates and survival capabilities are still unknown. However, using embryonic mosaicisms may result in an incorrect prediction of embryonic abnormalities, which would make establishing a consensus on mosaicism clinical application difficult [38]. Biological and technological limitations do exist and must be comprehended to adequately guide patients during clinical care since the dilemma remains on weather a mosaic embryo may result in a miscarriage or has the potential to develop into a viable, healthy baby. Nevertheless, the focus of this study was that if true euploid embryos were selected, then IVF results improved, independent of the source of the oocyte. Our current PGT system was able to determine mosaicisms when levels were > 30%, but not minor mosaicisms. Therefore, we did not assess embryonic mosaicisms with respect to IVF outcomes.

This study has a few limitations. First, this is a retrospective observational study. The analyses used in the study can show associations between variables but cannot establish causality. Second, the different qualities and quantities of hormones that were used during the ovary stimulation and IVF procedures within the 4-year timeframe could provide a source of bias. However, the heterogeneous nature of the sample (dose and response), which may lead a clinician to alter their course of treatment, was not the focus of the research. Many studies have demonstrated that these variations do affect the aneuploidy rate and should be taken into consideration. Nevertheless, the overall result that PGT should accompany IVF when donor oocytes are used is independent and supported by this concern. Lastly, only euploid embryos were implanted. Current suggested guidelines indicate that a certain level of aneuploidy embryos could be implanted and still produce a viable baby due to the embryos self-correcting mechanism [21]. This does suggest that, even for embryos from donor oocytes, the chromosome content should be determined.

## Conclusion

Here, we demonstrate that when euploid embryos are transferred, embryos derived from donor oocytes were similar to patient oocyte derived embryos with respect to IVF clinical outcomes: implantation, embryo development into clinical pregnancy, and live birth. Aneuploidy rates were more than 25% in donors, which means 1 of every 4 embryos from donor oocytes may not implant due to chromosome abnormalities. Therefore, it is highly recommended to complement IVF with PGT when donor oocytes are used.

## Supplementary information

**Additional file 1.** Prevalence of Aneuploidies in the cohort: Odds ratios (OR) and 95% confidence intervals were calculated using multinomial logistic regression, * indicates a significant result (p < 0.05, two-tailed), ** indicates a significant difference in the frequencies of chromosome abnormalities between the donor group and the ≤ 29 years old patient group (p < 0.05, two-tailed).

## Data Availability

The datasets used and/or analyzed during the current study are available from the corresponding author on reasonable request.

## Abbreviations

β-hCG: β-Human chorionic gonadotropin
aCGH: Array comparative genomic hybridization
CGH: Comparative genomic hybridization
IVF: In vitro fertilization
ICSI: Intracytoplasmic sperm injection
OR: Odds ratio
PGT: Pre-implantation Genetic Diagnosis testing
