# Supplementary material for: Embryos derived from donor or patient oocytes are not different for in vitro fertilization outcomes when PGT allows euploid embryo selection: a retrospective study
Source: Clin Transl Med. 2020 Feb 7;9:14. doi: 10.1186/s40169-020-0266-1 (PMC7005195; doi:10.1186/s40169-020-0266-1)
Supplement: Supplementary file 1 — Additional file 1. Prevalence of Aneuploidies in the cohort: Odds ratios (OR) and 95% confidence intervals were calculated using multinomial logistic regression, * indicates a significant result (p < 0.05, two-tailed), ** indicates a significant difference in the frequencies of chromosome abnormalities between the donor group and the ≤ 29 years old patient group (p < 0.05, two-tailed). [file 40169_2020_266_MOESM1_ESM.docx]

Supplemental Table. Prevalence of Aneuploidies in the cohort (Supplemental)

| Chr | Type | Total | ≤29 | 30-34 | 35-37 | 38-40 | 41-43 | >44 | Donors | OR ^a^ |
| --- | --- | --- | --- | --- | --- | --- | --- | --- | --- | --- |
|  |  | 3003 | 172 | 406 | 481 | 546 | 439 | 111 | 848 | 1 year increase |
| Chr_1 | Gain | 46 (1.5%) | 1 (0.6%) | 3 (0.7%) | 7 (1.5%) | 11 (2.0%) | 10 (2.3%) | 5 (4.5)% | 9 (1.1%) | 1.13 (1.04-1.23) * |
|  | Loss | 54 (1.8%) | 4 (2.3%) | 9 (2.2%) | 7 (1.5%) | 12 (2.2%) | 5 (1.1%) | 2 (1.8%) | 15 (1.8%) | 0.96 (0.90-1.02) |
|  |  |  |  |  |  |  |  |  |  |  |
| Chr_2 | Gain | 45 (1.5%) | 2 (1.2%) | 8 (2.0%) | 7 (1.5%) | 9 (1.6%) | 6 (1.4%) | 8 (7.2%) | 5 (0.6%) | 1.08 (1.00-1.16) |
|  | Loss | 43 (1.4%) | 2 (1.2%) | 4 (1.0%) | 12 (2.5%) | 7 (1.3%) | 4 (0.9%) | 3 (3.6%) | 10 (1.2%) | 1.01 (0.94-1.09) |
|  |  |  |  |  |  |  |  |  |  |  |
| Chr_3 | Gain | 37 (1.2%) | 1 (0.6%) | 1 (0.2%) | 7 (1.5%) | 9 (1.6%) | 4 (0.9%) | 4 (3.6%) | 11 (1.3%) | 1.15 (1.04-1.27) * |
|  | Loss | 20 (0.7%) | 0 (0.0%) | 2 (0.5%) | 4 (0.8%) | 2 (0.4%) | 5 (1.1%) | 2 (1.8%) | 5 (0.6%) | 1.13 (1.00-1.29) |
|  |  |  |  |  |  |  |  |  |  |  |
| Chr_4 | Gain | 46 (1.5%) | 1 (0.6%) | 5 (1.2%) | 12 (2.5%) | 10 (1.8%) | 8 (1.8%) | 3 (2.7%) | 7 (0.8%) | 1.04 (0.97-1.12) |
|  | Loss | 33 (1.1%) | 4 (2.3%) | 5 (1.2%) | 2 (0.4%) | 4 (0.7%) | 5 (1.1%) | 4 (3.6%) | 9 (1.1%) | 1.00 (0.92-1.09) |
|  |  |  |  |  |  |  |  |  |  |  |
| Chr_5 | Gain | 35 (1.2%) | 3 (1.7%) | 3 (0.7%) | 6 (1.2%) | 6 (1.1%) | 9 (2.1%) | 2 (1.8%) | 6 (0.7%) | 1.04 (0.96-1.13) |
|  | Loss | 40 (1.3%) | 2 (1.2%) | 8 (2.0%) | 8 (1.7%) | 4 (0.7%) | 6 (1.4%) | 4 (3.6%) | 8 (0.9%) | 1.00 (0.93-1.08) |
|  |  |  |  |  |  |  |  |  |  |  |
| Chr_6 | Gain | 62 (2.1%) | 3 (1.7%) | 8 (2.0%) | 21 (4.4%) | 11 (2.0%) | 7 (1.6%) | 2 (1.8%) | 10 (1.2%) | 0.99 (0.94-1.05) |
|  | Loss | 20 (0.7%) | 1 (0.6%) | 2 (0.5%) | 6 (1.2%) | 6 (1.1%) | 0 (0.0%) | 1 (0.9%) | 4 (0.5%) | 1.00 (0.91-1.11) |
|  |  |  |  |  |  |  |  |  |  |  |
| Chr_7 | Gain | 49 (1.6%) | 4 (2.3%) ** | 8 (2.0%) | 6 (1.2%) | 13 (2.4%) | 9 (2.1%) | 6 (5.4%) | 3 (0.4%) | 1.03 (0.97-1.10) |
|  | Loss | 23 (0.8%) | 1 (0.6%) | 1 (0.2%) | 3 (0.6%) | 4 (0.7%) | 2 (0.5%) | 4 (3.6%) | 8 (0.9%) | 1.13 (0.99-1.28) |
|  |  |  |  |  |  |  |  |  |  |  |
| Chr_8 | Gain | 31 (1.0%) | 1 (0.6%) | 1 (0.2%) | 9 (1.9%) | 8 (1.5%) | 6 (1.4%) | 2 (1.8%) | 4 (0.5%) | 1.07 (0.98-1.17) |
|  | Loss | 36 (1.2%) | 0 (0.0%) | 5 (1.2%) | 5 (1.0%) | 7 (1.3%) | 8 (1.8%) | 2 (1.8%) | 9 (1.1%) | 1.06 (0.97-1.15) |
|  |  |  |  |  |  |  |  |  |  |  |
| Chr_9 | Gain | 66 (2.2%) | 0 (0.0%) | 11 (2.7%) | 11 (2.3%) | 14 (2.6%) | 12 (2.7%) | 11 (9.9%) | 7 (0.8%) | 1.11 (1.04-1.18) * |
|  | Loss | 46 (1.5%) | 2 (1.2%) | 4 (1.0%) | 4 (0.8%) | 14 (2.6%) | 11 (2.5%) | 5 (4.5%) | 6 (0.7%) | 1.12 (1.04-1.21) * |
|  |  |  |  |  |  |  |  |  |  |  |
| Chr_10 | Gain | 54 (1.8%) | 1 (0.6%) | 11 (2.7%) | 8 (1.7%) | 8 (1.5%) | 14 (3.2%) | 7 (6.3%) | 5 (0.5%) | 1.08 (0.99-1.13) |
|  | Loss | 31 (1.0%) | 1 (0.6%) | 5 (1.2%) | 4 (0.8%) | 5 (0.9%) | 4 (0.9%) | 6 (5.4%) | 6 (0.7%) | 1.08 (0.98-1.18) |
|  |  |  |  |  |  |  |  |  |  |  |
| Chr_11 | Gain | 48 (1.6%) | 1 (0.6%) | 4 (1.0%) | 10 (2.1%) | 7 (1.3%) | 15 (3.4%) | 7 (6.3%) | 4 (0.5%) | 1.14 (1.06-1.24) * |
|  | Loss | 28 (0.9%) | 0 (0.0%) | 1 (0.2%) | 2 (0.4%) | 8 (1.5%) | 7 (1.6%) | 5 (4.5%) | 5 (0.6%) | 1.24 (1.11-1.39) * |
|  |  |  |  |  |  |  |  |  |  |  |
| Chr_12 | Gain | 52 (1.7%) | 1 (0.6%) | 5 (1.2%) | 5 (1.0%) | 12 (2.2%) | 14 (3.2%) | 7 (6.3%) | 8 (0.9%) | 1.16 (1.07-1.25) * |
|  | Loss | 28 (0.9%) | 0 (0.0%) | 2 (0.5%) | 3 (0.6%) | 6 (1.1%) | 8 (1.8%) | 4 (3.6%) | 5 (0.6)% | 1.21 (1.08-1.35) * |
|  |  |  |  |  |  |  |  |  |  |  |
| Chr_13 | Gain | 68 (2.3%) | 3 (1.7%) | 10 (2.5%) | 11 (2.3%) | 11 (2.0%) | 21 (4.8%) | 6 (5.4%) | 6 (0.7%) | 1.06 (1.00-1.13) * |
|  | Loss | 49 (1.6%) | 2 (1.2%) | 4 (1.0%) | 5 (1.0%) | 8 (1.5%) | 13 (3.0%) | 5 (4.5%) | 12 (1.4%) | 1.10 (1.01-1.18) * |
|  |  |  |  |  |  |  |  |  |  |  |
| Chr_14 | Gain | 53 (1.8%) | 1 (0.6%) | 3 (0.7%) | 9 (1.9%) | 16 (2.9%) | 7 (1.6%) | 8 (7.2%) | 9 (1.1%) | 1.14 (1.06-1.23) * |
|  | Loss | 44 (1.5%) | 1 (0.6%) | 5 (1.2%) | 4 (0.8%) | 11 (2.0%) | 11 (2.5%) | 2 (1.8%) | 10 (1.2%) | 1.08 (1.00-1.17) |
|  |  |  |  |  |  |  |  |  |  |  |
| Chr_15 | Gain | 92 (3.1%) | 2 (1.2%) | 6 (1.5%) | 11 (2.3%) | 21 (3.8%) | 34 (7.7%) | 9 (8.1%) | 9 (1.1%) | 1.17 (1.10-1.24) * |
|  | Loss | 76 (2.5%) | 1 (0.6%) | 3 (0.7%) | 13 (2.7%) | 30 (5.5%) | 15 (3.4%) | 9 (8.1%) | 5 (0.6%) | 1.15 (1.09-1.23) * |
|  |  |  |  |  |  |  |  |  |  |  |
| Chr_16 | Gain | 159 (5.3%) | 8 (4.7%) | 17 (4.2%) | 23 (4.8%) | 40 (7.3%) | 37 (8.4%) | 16 (14.4%) | 18 (2.1%) | 1.08 (1.04-1.13) * |
|  | Loss | 78 (2.6%) | 0 (0.0%) | 14 (3.4%) | 11 (2.3%) | 13 (2.4%) | 20 (4.6%) | 4 (3.6%) | 16 (1.9%) | 1.08 (1.02-1.14) * |
|  |  |  |  |  |  |  |  |  |  |  |
| Chr_17 | Gain | 52 (1.7%) | 1 (0.6%) | 4 (1.0%) | 5 (1.0%) | 8 (1.5%) | 18 (4.1%) | 7 (6.3%) | 9 (1.1%) | 1.19 (1.10-1.30) * |
|  | Loss | 20 (0.7%) | 0 (0.0%) | 3 (0.7%) | 0 (0.0%) | 4 (0.7%) | 7 (1.6%) | 3 (2.7%) | 3 (0.4%) | 1.18 (1.04-1.34) * |
|  |  |  |  |  |  |  |  |  |  |  |
| Chr_18 | Gain | 63 (2.1%) | 2 (1.2%) | 7 (1.7%) | 13 (2.7%) | 15 (2.7%) | 13 (3.0%) | 4 (3.6%) | 9 (1.1%) | 1.07 (1.00-1.13) * |
|  | Loss | 48 (1.6%) | 2 (1.2%) | 4 (1.0%) | 3 (0.6%) | 14 (2.6%) | 14 (3.2%) | 5 (4.5%) | 6 (0.7%) | 1.13 (1.05-1.22) * |
|  |  |  |  |  |  |  |  |  |  |  |
| Chr_19 | Gain | 115 (3.8%) | 3 (1.7%) | 23 (5.7%) | 14 (2.9%) | 33 (6.0%) | 24 (5.5%) | 6 (5.4%) | 12 (1.4%) | 1.04 (0.99-1.09) |
|  | Loss | 49 (1.6%) | 0 (0.0%) | 7 (1.7%) | 5 (1.0%) | 10 (1.8%) | 16 (3.6%) | 9 (8.1%) | 2 (0.2%) | 1.18 (1.09-1.27) * |
|  |  |  |  |  |  |  |  |  |  |  |
| Chr_20 | Gain | 85 (2.8%) | 1 (0.6%) | 8 (2.0%) | 10 (2.1%) | 18 (3.3%) | 21 (4.8%) | 15 (13.5%) | 12 (1.4%) | 1.19 (1.11-1.26) * |
|  | Loss | 47 (1.6%) | 1 (0.6%) | 9 (2.2%) | 2 (0.4%) | 10 (1.8%) | 14 (3.2%) | 6 (5.4%) | 5 (0.6%) | 1.13 (1.05-1.22) * |
|  |  |  |  |  |  |  |  |  |  |  |
| Chr_21 | Gain | 104 (3.5%) | 2 (1.2%) | 23 (5.7%) | 17 (3.5%) | 18 (3.3%) | 28 (6.4%) | 13 (11.7%) | 3 (0.4%) | 1.07 (1.03-1.12) * |
|  | Loss | 61 (2.0%) | 1 (0.6%) | 3 (0.7%) | 5 (1.0%) | 20 (3.7%) | 20 (4.6%) | 4 (3.6%) | 8 (0.9%) | 1.14 (1.07-1.22) * |
|  |  |  |  |  |  |  |  |  |  |  |
| Chr_22 | Gain | 115 (3.8%) | 3 (1.7%) | 12 (3.0%) | 16 (3.3%) | 42 (7.7%) | 22 (5.0%) | 12 (10.8%) | 8 (0.9%) | 1.10 (1.05-1.15) * |
|  | Loss | 100 (3.3%) | 3 (1.7%) | 2 (0.5%) | 14 (2.9%) | 23 (4.2%) | 39 (8.9%) | 11 (9.9%) | 8 (0.9%) | 1.20 (1.14-1.28) * |
|  |  |  |  |  |  |  |  |  |  |  |
| Chr_X | Gain | 70 (2.3%) | 4 (2.3%) ** | 10 (2.5%) | 13 (2.7%) | 15 (2.7%) | 18 (4.1%) | 4 (3.6%) | 6 (0.7%) | 1.04 (0.99-1.10) |
|  | Loss | 50 (1.7%) | 3 (1.7%) | 5 (1.2%) | 12 (2.5%) | 12 (2.2%) | 5 (1.1%) | 3 (2.7%) | 10 (1.2%) | 1.02 (0.96-1.10) |
|  |  |  |  |  |  |  |  |  |  |  |
| Chr_Y | Gain | 16 (0.5%) | 3 (1.7%) | 1 (0.2%) | 4 (0.8%) | 3 (0.5%) | 1 (0.2%) | 0 (0.0%) | 4 (0.5%) | 0.91 (0.82-1.01) |
|  | Loss | 89 (3.0%) | 2 (1.2%) | 10 (2.5%) | 16 (3.3%) | 16 (2.9%) | 10 (2.3%) | 6 (5.4%) | 29 (3.4%) | 1.05 (0.99-1.11) |

^a^ Odds ratios (OR) and 95% confidence intervals were calculated using multinominal logistic regression. * indicates a significant result (p<0.05, two-tailed).

** indicates a significant difference in the frequencies of the chromosome abnormality between the donor group and the ≤29-year-old age group (p<0.05, two-tailed).
